# Regulation of epigenetic traits of the glutathione *S*-transferase P1 gene: from detoxification toward cancer prevention and diagnosis

**DOI:** 10.3389/fphar.2014.00170

**Published:** 2014-07-16

**Authors:** Michael Schnekenburger, Tommy Karius, Marc Diederich

**Affiliations:** ^1^Laboratoire de Biologie Moléculaire et Cellulaire du Cancer, Hôpital KirchbergLuxembourg; ^2^College of Pharmacy, Seoul National University, SeoulSouth Korea

**Keywords:** GSTP1, cancer, epigenetics, DNA methylation, histone modifications, epimutations, biomarker

## Abstract

Glutathione *S*-transferases (GSTs) are phase II drug detoxifying enzymes that play an essential role in the maintenance of cell integrity and protection against DNA damage by catalyzing the conjugation of glutathione to a wide variety of exo- and endogenous electrophilic substrates. Glutathione *S*-transferase P1 (GSTP1), the gene encoding the pi-class GST, is frequently inactivated by acquired somatic CpG island promoter hypermethylation in multiple cancer subtypes including prostate, breast, liver, and blood cancers. Epigenetically mediated GSTP1 silencing is associated with enhanced cancer susceptibility by decreasing its “caretaker” gene function, which tends to promote neoplastic transformation allowing cells to acquire additional alterations. Thus, this epigenetic alteration is now considered as a cancer biomarker but could as well play a driving role in multistep cancer development, especially well documented in prostate cancer development. The present review discusses applications of epigenetic alterations affecting GSTP1 in cancer medicine used alone or in combination with other biomarkers for cancer detection and diagnosis as well as for future targeted preventive and therapeutic interventions including by dietary agents.

## INTRODUCTION

As one of the driving forces behind the cellular detoxification machinery, glutathione *S*-transferases (GSTs) and especially the pi class glutathione *S*-transferase P1 (GSTP1) is currently in the focus of the cancer research community, evaluating the relevance of GSTP1 epimutations for cancer development and its potential as a major epigenetic cancer biomarker.

The human GST multi-gene superfamily is encoding for various ubiquitous cytosolic or soluble, mitochondrial and microsomal as well as peroxisomal homo- and heterodimeric transferases (Di Pietro et al., 2010). Despite the multifunctionality of these proteins, GSTs are best known for their ability to transfer the tripeptide gamma-glutamyl-cysteinyl-glycine, also known as glutathione (GSH) to a wide variety of highly genotoxic and cell-damaging molecules, either directly occurred from the extracellular environment or from the intracellular detoxification metabolism. In most albeit not all cases, glutathione *S*-conjugation generates a less or non-toxic product with improved water solubility, favoring the exportation out of the cell and thereby contributing to DNA damage prevention and protection of the cellular integrity (Baden et al., 2011). Moreover, several GST isozymes are implicated in cell signaling, interfering for example with the MAPK signaling cascade, which is involved in the regulation of cell cycle, proliferation and cell death (Wang et al., 2001; Laborde, 2010).

Regardless the importance of GST activity for cellular vitality and health, the GST gene cluster is a hotspot for DNA sequence mutations that leads to the expression of active but functionally different GST variant proteins. Accordingly, cells expressing less active GST isoforms are more sensible to GST-metabolized toxins compared to cells with balanced GST activity. In a worst case-scenario, cells are incapable to degrade carcinogens or stress-induced toxic intermediates, thus increasing their susceptibility to undergo further steps toward cancer progression or event other diseases (Deep et al., 2012). The GSTP1^∗^B (Val105) allele is often mentioned within the context of genetic polymorphisms, a GSTP1 variation which is characterized by an A→G sequence transition in codon 105 of exon 5, leading to the exchange of isoleucine by valine, thus decreasing its catalytic activity associated with reduced cell detoxification ability (Saxena et al., 2012).

## IMPORTANCE OF GLUTATHIONE *S*-TRANSFERASE P1 CLASS IN CANCER DEVELOPMENT

**FIGURE 1:**
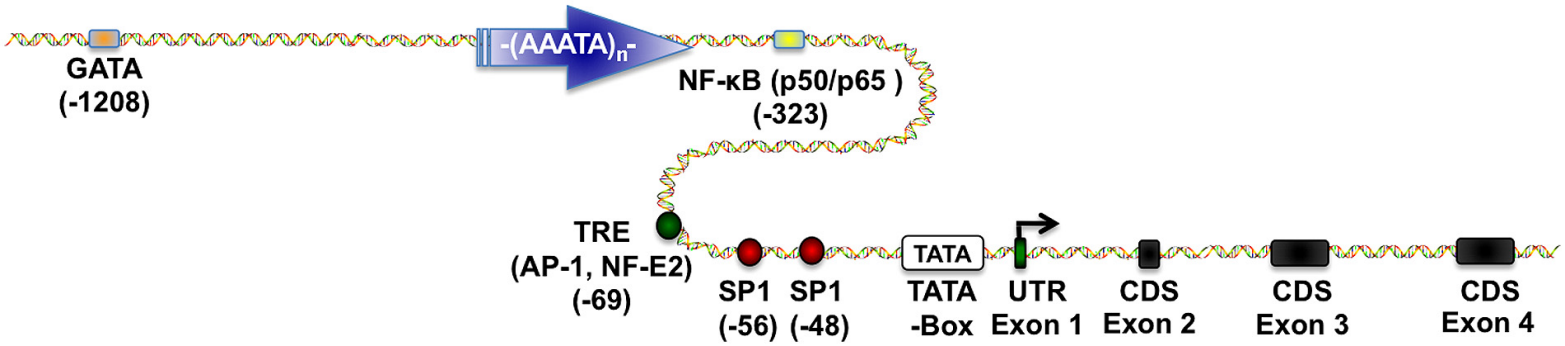
**Glutathione *S*-transferase P1 (GSTP1) regulatory elements.** This scheme depicts essential transcriptional regulatory elements known to regulate GSTP1 gene expression. Proximal promoter region contains (i) two SP1 sites (Morrow et al., 1989), (ii) one TPA-response element (TRE) binding (activator protein) AP-1 and nuclear factor, erythroid 2 (NF-E2) depending on the cellular environment (Borde-Chiche et al., 2001b; Duvoix et al., 2003a,b, 2004a,b), (iii) one nuclear factor kappa-light-chain-enhancer of activated B cells (NF-κB) binding site (Morceau et al., 2004), and (iv) a GATA-1 binding site (Schnekenburger et al., 2003, 2004). Figure was generated by using ScienceSlides.

Furthermore, the previously mentioned cytosolic GSTP1 isoenzyme consist of one of the best-studied variants of the GST metabolism. Located on chromosome 11, the GSTP1 coding region is controlled by a large CpG island (CGI) upstream of the transcription start site in the promoter region. Both areas are separated by a long ATAAA repetitive stretch, which probably acts as an insulator to separate different epigenetic states such as methylation of the CGIs (Millar et al., 2000). Moreover, various transcription factors such as specificity protein 1 (SP1), activator protein 1 (AP-1), nuclear factor kappa-light-chain-enhancer of activated B cells (NF-κB) and GATA1 were reported to play an important role in the regulation of GSTP1 expression (**Figure 1**; Moffat et al., 1996; Duvoix et al., 2003b, 2004b; Schnekenburger et al., 2003; Morceau et al., 2004).

Glutathione *S*-transferase P1 is also involved in cell death regulation, interacting with apoptotic signaling pathways as for example c-Jun NH2-terminal kinase (JNK1), ERK1/ERK2, or tumor necrosis factor receptor-associated factor 2 (TRAF2; Ruscoe et al., 2001; Wu et al., 2006). In correlation to its biotransformation and detoxification ability, GSTP1 is expressed in most cells and particularly in those that are in contact with the external environment such as cells of the urinary, digestive, and respiratory tract (Terrier et al., 1990; Yuan et al., 2008).

Increased levels of GSTP1 expression can also be indicative for enhanced detoxification activity due to xeno- or endo-biotic exposure implicating oxidative stress (Kanwal et al., 2014). Accordingly, increased GSTP1 expression is often detected in many cancers (e.g., breast, colon, stomach, pancreas, bladder, lung, head and neck, ovary and cervix, soft tissue sarcoma, testicular embryonic carcinoma, meningioma, and glioma), which is associated to enhanced detoxification activity, thus protecting cancer cells against cytotoxic and cytostatic drugs (Ruzza et al., 2009). In contrast, knockout experiences in mice showed that loss of GSTP1 expression leads to increased cancer susceptibility (Henderson et al., 1998; Ketterer, 1998).

## REGULATION OF CELLULAR ACTIVITIES THROUGH EPIGENETIC MECHANISMS

Alterations in the epigenetic setup are as important as genetic aberrations and may cooperate in cancer genesis. While classical gene mutations are region-limited, epimutations often occur early in cancer development and have a genome-wide impact, boosting on the one hand the expression of cell survival genes or proto-oncogenes and deactivating on the other hand tumor suppressor genes (TSGs), DNA repair mechanisms as well as cell division brakes, thus leading to carcinogenesis (Esteller, 2008; Florean et al., 2011; Karius et al., 2012; Schnekenburger and Diederich, 2012; Seidel et al., 2012; Blancafort et al., 2013).

In addition to the genetic information encoded by the primary DNA sequence, epigenetic mechanisms add a layer of regulation of the information and includes DNA methylation, histone modifications as well as regulation by non-coding RNAs (Esteller, 2008; Florean et al., 2011; Karius et al., 2012; Schnekenburger and Diederich, 2012; Seidel et al., 2012; Blancafort et al., 2013). This interacting cluster of epigenetic regulators provides an epigenetic memory, transferring epigenetic information through mitotic and meiotic cell divisions (Migicovsky and Kovalchuk, 2011). These reversible modifications are playing essential roles in gene regulation, X-inactivation, imprinting, and silencing of parasitic DNA elements (Jaenisch and Bird, 2003).

### PATHOLOGICAL ALTERATIONS OF GSTP1 METHYLATION PATTERNS

DNA methylation, the addition of a methyl-group to cytosines is the most common epigenetic modification, inducing the reorganization of the gene locus and thus regulating gene expression (Smith and Meissner, 2013). However, tumor cells typically possess an aberrant methylation pattern associated with altered gene expression profiles, showing locally restricted hypermethylation of individual promoter regions involved in the silencing of TSGs. At the same time, genome-wide loss of DNA methylation is observed in tumor cells compared to healthy cells inducing chromosomal instability, loss of imprinting as well as the previously described oncogene activation.

**FIGURE 2:**
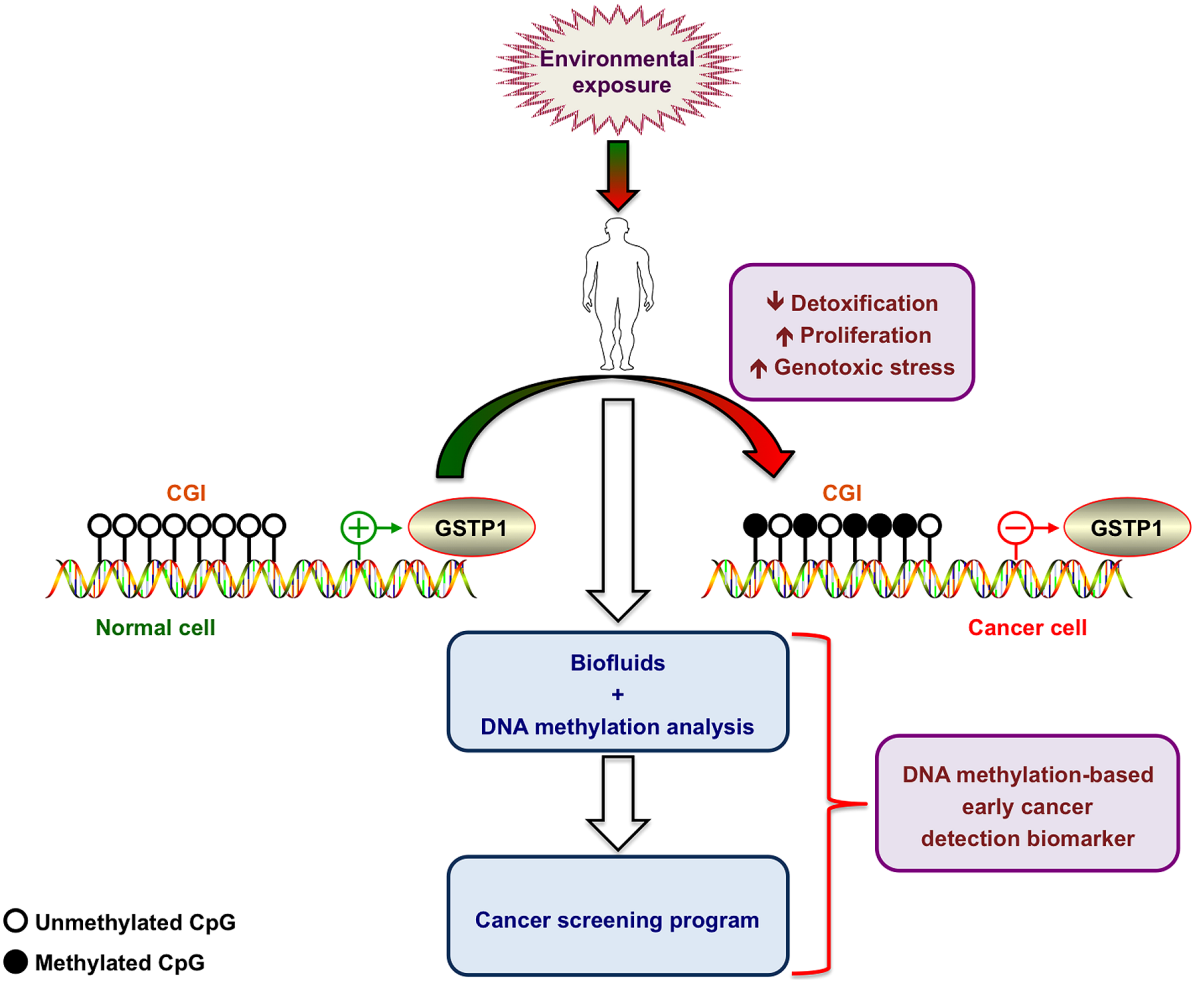
**Hypermethylation of the glutathione *S*-transferase P1 promoter as early cancer biomarker.** Environmental exposure leads to progressive methylation of CpG islands (CGI) at the GSTP1 promoter region. The resulting decreased cell detoxification capacity leads to increased genotoxic stress and progressive accumulation of additional genetic alterations accompanied by an increased proliferation rate. DNA methylation analysis of the GSTP1 promoter in body fluids allows detecting progressive hypermethylation and turns this molecular feature into a valid early cancer biomarker. Figure was generated by using ScienceSlides.

In its function as a cellular “caretaker”, attenuation of GSTP1 expression or activity by either genetic (e.g., deletion or mutation) or epigenetic alterations may reduce cellular detoxification capacity (**Figure 2**). In fact, the use of GSTP1 knockout mice demonstrated that loss of GSTP1 expression increases sensitivity to metabolic or environmental toxins and promotes mutations and cancer development (Coughlin and Hall, 2002a,b).

#### Prostate cancer

Promoter hypermethylation leading to epigenetic silencing of GSTP1 gene expression is frequently detected in prostate cancer cells, the most commonly diagnosed type of malignancy among men in Western European countries and the second cause of cancer-related deaths among men worldwide (Brooks et al., 1998; Cairns et al., 2001; Jeronimo et al., 2002; Henrique and Jeronimo, 2004; Dumache et al., 2010; Ferlay et al., 2010). Interestingly, GSTP1 hypermethylation is strictly restricted to malignant cells including prostate cancer cells (PCa) as well as prostatic intraepithelial neoplasia (PIN). Detection of GSTP1 methylation in all types of body fluids of prostate cancer patients represents a promising epigenetic biomarker, which is already under evaluation for the application of new prognostic methods (Cairns et al., 2001; Esteller, 2008; Yang and Park, 2012; **Figure 2**). In contrast, GSTP1 promoter remains almost unmethylated in benign lesions, allowing distinguishing benign and cancerous transformations (Hopkins et al., 2007; Cao and Yao, 2010). Recently, Re et al. (2011) published that, beyond hypermethylation, chromatin remodeling by a combinatorial complex between estrogen receptor (ER) and endothelial nitric oxide synthase (eNOS) also represses transcription of prognostic genes that are down-regulated in PCa, such as GSTP1. In PCa cultured cells ER/eNOS causes GSTP1 repression by being recruited at estrogen responsive elements within its promoter favoring a local chromatin remodeling together with hypermethylated promoter sequences.

#### Lung cancer

Esteller et al. (1999) published that aberrant hypermethylation of the TSG p16, the putative metastasis suppressor gene death-associated protein kinase (DAPK), GSTP1, and the DNA repair gene O6-methylguanine-DNA-methyltransferase (MGMT) is observed in non-small cell lung cancer (NSCLC) tumors but not in any paired normal lung tissue. In primary tumors with methylation, 11 of 15 (73%) samples also had abnormal methylated DNA in the matched serum samples. More recently, Kim et al. (2005) concluded that DNA methylation status of CGI could be used as a predictor of long-term outcome for adenocarcinoma of the lung.

#### Leukemia and lymphoma

Borde-Chiche et al. (2001a) published that methylation of CpG sites of the basal GSTP1 promoter is an essential mechanism controlling GSTP1 gene expression in human leukemia. Karius et al. (2011) further investigated this mechanism and showed by bisulfite sequencing, methylation-specific PCR and combined bisulfite restriction analysis that the GSTP1 promoter was completely methylated in transcriptionally inactive in RAJI Burkitt’s lymphoma and MEG-01 chronic myeloid leukemia cell lines. In contrast, cell lines expressing GSTP1 exhibited an unmethylated and transcriptionally active promoter thus confirming a relationship between hypermethylation and repression of GSTP1 expression (Karius et al., 2011).

Rossi et al. (2004) investigated methylation of MGMT, DAPK, and GSTP1 and concluded that these alterations represent a major pathogenetic event in several B-cell malignancies. Inactivation of GSTP1 in gastric MALT lymphoma represents an additional mechanism favoring accumulation of reactive oxygen species to further promote lymphomagenesis. Finally, frequency of GSTP1 aberrant methylation in diffuse large B-cell lymphoma (DLBCL) also led to studies to validate the prognostic impact of such epigenetic alteration in these lymphomas. Nakamichi et al. (2007) established a correlation between promoter hypermethylation of GSTP1 and response to chemotherapy in DLBCL. According to the authors, the GSTP1 gene methylation status could be an indicator of drug response and a prognosticator for DLBCL (Nakamichi et al., 2007).

#### Breast cancer

Jhaveri and Morrow initially published that methylation status of GSTP1 promoter contributes significantly to the levels of GSTP1 expressed in ER-negative and ER+-positive breast cancer cell lines (Jhaveri and Morrow, 1998). Moreover, GSTP1 hypermethylation and therefore gene silencing was associated to increased grades of mammary phyllodes tumors. As such GSTP1 methylation patterns allow distinguishing two groups: one benign unmethylated group as well as samples presenting hypermethylated GSTP1 gene promoters in the borderline/malignant tumor group (Kim et al., 2009). Recently, Miyake et al. (2012) demonstrated that GSTP1 expression predicts poor pathological complete response to neoadjuvant chemotherapy in ER-negative breast cancer. Indeed, GSTP1 expression can predict pathological response to chemotherapeutic treatments with 5-fluorouracil/epirubicin/cyclophosphamide in ER-negative tumors but not in ER-positive tumors. Additionally, GSTP1 promoter hypermethylation might be implicated more importantly in the pathogenesis of luminal A, luminal B, and HER2-enriched tumors, than in basal-like tumors.

#### Liver cancer

According to Zhang et al. (2012) GSTP1 is transcriptionally silenced by promoter hypermethylation in several human cancer types including hepatocellular carcinoma (HCC). These results suggest that epigenetic inactivation of GSTP1 plays an important role in the development of HCC and exposure to environmental carcinogens may be related to altered methylation of genes involved in hepatocarcinogenesis (Zhang et al., 2012).

#### Other cancer subtypes

Similarly, the invasion potential of pituitary tumors and endometrial carcinomas was linked to reduction of GSTP1 expression and methylation frequency, indicating that epigenetically mediated down-regulation of GSTP1 expression may also contribute to aggressive pituitary tumor behavior (Chan et al., 2005; Yuan et al., 2008).

### PATHOLOGICAL ALTERATIONS OF GSTP1 METHYLATION PATTERNS

Beyond DNA hypermethylation, which was the first epigenetic alteration to be discovered as an influencing factor for GSTP1 expression, histone modifications were discovered in the early 2000 s to play a regulating role in GSTP1 expression. Moreover, an interplay between histone reprogramming and DNA methylation was emerging. Bakker et al. (2002) initially showed that methyl-CpG binding domain protein (MBD)2 represses transcription from hypermethylated GSTP1 gene promoters in HCC cells connecting hypermethylation and reduced transactivation potential. Similarly, Lin and Nelson (2003) published that MDB2 mediates transcriptional repression associated with hypermethylated GSTP1 CGIs in MCF-7 breast cancer cells.

HATs (histone acetyltransferases) contribute to the regulation of gene expression, and loss or deregulation of these activities may link to tumorigenesis. Ohta et al. (2007) demonstrated that expression levels of HATs, p300, and CBP [CREB (cAMP-response-element-binding protein)-binding protein] were decreased during chemical hepatocarcinogenesis, whereas expression of MOZ (monocytic leukemia zinc-finger protein; MYST3), a member of the MYST [MOZ, Ybf2/Sas3, Sas2, and TIP60 (Tat-interacting protein, 60 kDa)] HAT family was induced. Exogenous MOZ induced GSTP1 expression in rat hepatoma H4IIE cells. These results suggest that during early hepatocarcinogenesis, aberrantly expressed MOZ may induce GSTP1 expression through the NF-E2-related factor 2 (Nrf2)-mediated pathway.

Okino et al. (2007) investigated chromatin changes on GSTP1 promoter associated with its inactivation in prostate cancer. Thus, treatment of LNCaP cells with the DNA demethylating agent 5-azacytidine also restored activating histone modifications on GSTP1 and as a result reactivated transcription. Authors concluded that, in the process of prostate carcinogenesis, activating histone modifications on GSTP1 are lost and subsequently DNA becomes methylated and inaccessible resulting in transcriptional silencing thus demonstrating interplay between histone reprogramming and promoter region methylation (Okino et al., 2007).

Karius et al. (2011) further contributed that histone marks and effector proteins associated with transcriptional activity could be detected by chromatin immunoprecipitation in GSTP1 expressing hypomethylated K-562 cell line. However, repressive chromatin marks and the recruitment of silencing protein complexes were found in the non-expressing hypermethylated RAJI and MEG-01 cell lines, again validating the interrelationship between DNA methylation and histone marks (Karius et al., 2011).

In prostate cancer cells, Hauptstock et al. (2011) used the histone deacetylase inhibitor depispeptide to reverse DNA hypermethylation and alter the histone modification pattern at GSTP1 promoter, including a reduction of H3K9me2/3 and H3K27me2/3 and an increase of H3K18Ac, thereby inducing GSTP1 mRNA re-expression. For these authors, successful therapy requires both, DNA demethylation and triggering activating histone modifications, to induce complete gene expression of epigenetically silenced genes and depsipeptide fulfills both criteria (Hauptstock et al., 2011).

### SMALL REGULATORY RNA INVOLVED IN GSTP1 EXPRESSION

MicroRNAs (miRNAs) constitute a large family of regulatory non-coding (nc)RNAs (Morceau et al., 2013). These single stranded RNAs (17–25 nucleotides) are highly conserved during evolution and are generated by a multistage process. Their expression leads to post-transcriptional silencing of target genes by mRNA translation repression. MiRNAs target several mRNAs while a specific mRNA can be targeted by several miRNAs. Altogether these regulatory mechanisms also belong to epigenetic regulation.

Accumulating evidence suggest that, as most mRNAs, the level of GSTP1 transcripts can potentially be regulated by several miRNAs. Patron et al. (2012) published that miR-133b reduces GSTP1 expression by 2.1 fold in prostate cancer cells. In addition, miR-513a-3p sensitizes human A549 lung adenocarcinoma cells to chemotherapy by targeting GSTP1 (Zhang et al., 2012). Mutallip et al. (2011) showed that transient transfection of miR-133a repressed the expression of GSTP1 at mRNA and protein levels. Similar results were published in human bladder cancer (Uchida et al., 2013) and earlier in lung squamous cell carcinoma (Moriya et al., 2012).

Even though only limited information is available regarding the regulation of GSTP1 by miRNAs, we believe that further investigations could contribute to the development of new therapeutic miRNA-based anticancer strategies.

## DIETARY REGULATORS OF GSTP1 EXPRESSION

Since progressive GSTP1 hypermethylation is a hallmark biomarker but potentially also a driver of prostate cancer progression, various research teams were looking for dietary intervention to lower the methylation burden of GSTP1 gene promoter. By re-expressing GSTP1, increased detoxification, and reduced levels of oxidative stress could potentially contribute to reduced prostate cancer progression and even to an abrogation on evolution toward invasive and metastatic disease.

Accordingly, many studies reported dietary nutrients or phytochemicals that present the potential to restore GSTP1 expression (Schnekenburger et al., 2014). For instance, Vardi et al. (2010) showed that soy phytoestrogens modify DNA methylation of GSTP1, RASSF1A, EPH2, and BRCA1 TSG promoters in prostate cancer cells. After treatment by phytoestrogens, demethylation of GSTP1, and EPHB2 promoter regions was observed and an increase in their protein expression levels was demonstrated by immunohistochemistry. Altogether epigenetic modifications of DNA, such as the promoter CGI demethylation of TSGs, might be related to the protective effect of soy on prostate cancer (Vardi et al., 2010). In prostate cancer cells, phenethyl isothiocyanate, a phytochemical found in large amounts in cruciferous vegetables, was reported to restore expression of silenced GSTP1 by a mechanism involving promoter demethylation and increased histone acetylation. These effects are associated with increased expression of the cyclin-dependent kinase inhibitors (CDKNs) p21 and p27, which are negative cell cycle regulators (Huang et al., 2011). Furthermore, it was established that GSTP1 gene was demethylated and reactivated following exposure to green tea polyphenols in prostate cancer cells (Pandey et al., 2010). Interestingly, lycopene also reactivated GSTP1 gene expression through reduced promoter methylation in MDA-MB-468 breast cancer cells (King-Batoon et al., 2008). In rodents, choline deficiency results in global hypomethylation of hepatic DNA and aberrant DNA hypermethylation at targeted TSG promoters such as of the GSTP1 gene promoter (Zeisel, 2012). Finally, Xiang et al. (2008) published that selenite (Se) treatment decreased general DNA methylation and caused partial promoter demethylation and re-expression of the TSGs adenomatous polyposis coli (APC) and cellular stress response 1, a gene involving tumor growth and metastasis. This study demonstrates that Se can epigenetically modulate DNA and histones to activate methylation-silenced genes. These epigenetic modifications may altogether contribute to cancer prevention by Se (Xiang et al., 2008).

## CONCLUSIVE REMARKS

Considering that the discrimination power of serum prostate-specific antigen (PSA) measurement between benign and malignant tumor cells is currently under controversial discussion, the best and the most promising epigenetic marker for prostate cancer detection is the hypermethylation of GSTP1.

The previously mentioned specificity for prostate intraepithelial neoplasia allows differentiating from unmethylated benign hyperplastic prostate tissue, which always remains unmethylated. Moreover, many tumors including PCa cells shed DNA into the serum or other easily accessible body fluids (e.g., semen, urine), simplifying the detection of GSTP1 epimutations in early tumorigenesis stages. Indeed, early presence of hypermethylated TSGs does not necessarily indicate an invasive cancer, as premalignant or cancer precursor lesions can also carry these epigenetic signatures. Hence, these signatures, including miRNAs, could be used for early cancer detection in individuals with genetic predispositions or exposed to carcinogens.

## Conflict of Interest Statement

The authors declare that the research was conducted in the absence of any commercial or financial relationships that could be construed as a potential conflict of interest.
